# Investigating the potential association of temporary employment and job dissatisfaction with alcohol use disorder and depressive symptoms: a 13-wave longitudinal analysis

**DOI:** 10.1192/bjo.2023.40

**Published:** 2023-04-12

**Authors:** Seong-Uk Baek, Jin-Ha Yoon, Jong-Uk Won

**Affiliations:** Department of Occupational and Environmental Medicine, Severance Hospital, Yonsei University College of Medicine, Korea; The Institute for Occupational Health, Yonsei University College of Medicine, Korea; and Graduate School, Yonsei University College of Medicine, Korea; The Institute for Occupational Health, Yonsei University College of Medicine, Korea; Graduate School of Public Health, Yonsei University College of Medicine, Korea; and Department of Preventive Medicine, Yonsei University College of Medicine, Korea; Department of Occupational and Environmental Medicine, Severance Hospital, Yonsei University College of Medicine, Korea; The Institute for Occupational Health, Yonsei University College of Medicine, Korea; and Graduate School of Public Health, Yonsei University College of Medicine, Korea

**Keywords:** Job insecurity, mental health, health behaviours, psychiatric distress, alcohol misuse

## Abstract

**Background:**

There has been growing interest in protecting workers’ mental health. Identifying social determinants that affect workers’ mental health could play an important role in preventing psychiatric diseases.

**Aims:**

We investigated the effects of temporary employment and job dissatisfaction on alcohol use disorder and depressive symptoms.

**Method:**

The Korea Welfare Panel Study data-set (2009–2021) was used, and 9611 participants with 52 639 observations were included. Generalised linear mixed models were employed to estimate odds ratios and 95% confidence intervals. The relative excess risk due to interaction (RERI) was calculated to assess supra-additive interactions between temporary employment and job dissatisfaction.

**Results:**

Increased risks for depressive symptoms were observed among fixed-term workers (odds ratio 1.12, 95% CI 1.00–1.26) and daily labourers (odds ratio 1.68, 95% CI 1.44–1.95). Daily labourers were associated with an increased risk of alcohol use disorder (odds ratio 1.54, 95% CI 1.22–1.95). Job dissatisfaction was associated with alcohol use disorder (odds ratio 1.78, 95% CI 1.52–2.08) and depressive symptoms (odds ratio 4.88, 95% CI 4.36–5.46). This effect became stronger when workers were concurrently exposed to temporary employment and job dissatisfaction. Daily labourers with job dissatisfaction showed the highest risks for alcohol use disorder (odds ratio 2.99, 95% CI 2.21–4.03) and depressive symptoms (odds ratio 9.00, 95% CI 7.36–11.02). RERIs between daily employment and job dissatisfaction were >0 for alcohol use disorder (0.91, 95% CI 0.06–1.76) and depressive symptoms (3.47, 95% CI 1.80–5.14), indicating a supra-additive interaction.

**Conclusions:**

We revealed that temporary employment and job dissatisfaction had detrimental effects on alcohol use disorder and depressive symptoms.

In the past few decades, changes in industrial structure and economic crises have led to an increase in the proportion of employment contracts other than standard employment.^1^ These non-standard types of employment contracts comprise temporary employment, part-time employment, disguised employment and employment arrangement via digital platforms.^2^ In particular, the informalisation of employment was accelerated by the recent COVID-19 pandemic. The COVID-19 pandemic caused an economic crisis and brought changes to the industrial structure, resulting in companies increasing their proportion of non-standard employment.^3^

## Temporary employment

Temporary employment is widely known for its negative effects on workers’ health. For example, workers with temporary employment are at risk of fatal occupational injuries^4^ and musculoskeletal problems.^5^ So far, several studies have investigated the effects of temporary employment on workers' mental health. Temporary employment can cause mental health problems because of the resulting subjective job insecurity for workers.^6^ Previous studies found that temporary employment is associated with psychiatric distress and depression.^7,8^ Kim et al reported that daily labourers had a higher risk of depressive symptoms compared with fixed-term or permanent workers in Korea.^9^ Furthermore, one previous study argued that temporary employment is associated with a higher risk of suicidal ideation.^10^ However, only a few studies have examined the relationship between temporary employment and risky health behaviours such as alcohol consumption. Although some researchers maintained that temporary employment was associated with alcohol misuse^11^ and increased mortality owing to alcohol-related diseases,^12^ others found no significant relationship between alcohol use and temporary employment.^13^

## Job dissatisfaction

Along with temporary employment, job satisfaction is also a major determinant of workers’ mental health. Job dissatisfaction is a well-known cause of burnout^14^ and reduced well-being^15^ of workers. Previous longitudinal and meta-analytic studies have reported that decreased job satisfaction is closely related to the poor mental health of workers.^16,17^ For example, cumulative years of job dissatisfaction were found to be associated with depressive symptoms^18^ and anxiety.^19^ On the other hand, the effect of job dissatisfaction on alcohol use is not unknown. Although one cross-sectional study found an association between job dissatisfaction and alcohol misuse among young male workers,^20^ another study found that job dissatisfaction did not mediate the relationship between work stress and alcohol use disorder.^21^

## Association of temporary employment and job dissatisfaction with mental health problems

Recently, there has been growing social interest in protecting workers’ mental health. Alcohol use disorder and depression are major causes of suicidal thoughts/behaviour, and can induce various physical illnesses such as liver diseases and obesity.^22^ Impaired mental health is harmful not only to workers themselves, but also to their organisations, as problematic alcohol consumption and depression negatively affect an individual's work performance.^23,24^

Currently, there are no studies that focus on the importance of job satisfaction for mental health problems in the context of temporary employment. In general, temporary employment has been reported to be closely correlated with low job dissatisfaction.^25^ Nevertheless, the relationship between temporary employment and job satisfaction depends on the characteristics of the worker. For instance, a decrease in job satisfaction was observed only in individuals who worked involuntarily in temporary employment, and in the case of voluntary temporary workers, no significant relationship between employment type and job satisfaction was found.^26^ Also, an interactional analysis by Hammarström et al. found that the health effect of temporary employment varied according to individual characteristics, such as education level.^7^ Thus, it could be hypothesised that the impact of temporary employment on the mental health of workers could be moderated by job satisfaction. Consequently, identifying how temporary employment and job dissatisfaction interact would provide new insight into the effect of temporary employment on workers’ mental health.

To sum up, the effect of temporary employment and job dissatisfaction on alcohol use disorder has not yet been identified. In addition, to the best of our knowledge, the combined effect of temporary employment and job dissatisfaction has not yet been reported. Therefore, our study will contribute to the literature by exploring the association of temporary employment and job dissatisfaction with alcohol use disorder and depressive symptoms, using a longitudinal data-set consisting of a nationally representative study sample. Finally, we aimed to discover social determinants affecting workers’ mental health, and provide evidence that could be used for policy development.

## Method

### Study sample

This study used the Korea Welfare Panel Study (KoWePS) data-set. The KoWePS is an ongoing annual panel survey conducted by the Seoul National University Social Welfare Research Centre and the Korea Institute for Health and Affairs since 2006. To collect nationally representative households in Korea, the KoWePS used two-stage stratified cluster sampling based on the South Korea Population and Housing Census. In this sampling method, the census enumeration district was employed as the primary sampling unit, and the household was employed as the secondary unit. The survey was conducted by trained interviewers using one-on-one computer-assisted personal interviews.

As the questionnaire regarding alcohol use disorder was introduced in 2009, study participants from 2009 to 2021 were included in this study. Out of a total of 23 692 participants with 204 336 observations, we limited our sample to those aged between 19 and 64 years because 65 years is the retirement age in Korea, leaving 14 703 participants with 107 591 observations. Next, we limited our sample to employed workers, leaving 9922 participants with 55 770 observations. After excluding 3131 observations with missing values, our final sample included 9611 participants with 52 639 observations.

### Ethics statement

The Institutional Review Board (IRB) of Yonsei Health System approved this study (approval number: 4-2022-0793). The requirement of informed consent was waived by the IRB because of the retrospective nature of this study.

### Main variables

#### Employment type

Employment types were classified as permanent workers, fixed-term workers and daily labourers. Permanent workers were employees who worked under a labour contract period of ≥1 year. Fixed-term workers were employees who worked under a labour contract period of ≥1 month to <1 year. Daily labourers were employees with a labour contract period of <1 month or those who were paid daily.

#### Job satisfaction

Job satisfaction was measured on a five-point Likert scale (1 = ‘very dissatisfied’, 2 = ‘fairly dissatisfied’, 3 = ‘neither satisfied nor dissatisfied’, 4 = ‘fairly satisfied’ and 5 = ‘very satisfied’). We then classified workers’ job satisfaction as ‘dissatisfied’ (fairly dissatisfied or very dissatisfied), ‘neutral’ (neither satisfied nor dissatisfied) or ‘satisfied’ (fairly satisfied or very satisfied).

#### Alcohol use disorder

The Alcohol Use Disorders Identification Test (AUDIT) was used to classify alcohol use disorders in this study. The ten-item AUDIT developed by the World Health Organization^27^ is a validated screening tool for identifying individuals with alcohol use disorder and has been widely used in epidemiologic studies. Each question is measured on a five-point Likert scale ranging from 0 to 4, so the total scores ranged from 0 to 40. According to previous studies, those with a score of ≥16 were considered to have a high level of alcohol problems and required clinical intervention and monitoring.^27^ Thus, the same value was used in this study for classifying those who had alcohol use disorder.

#### Depressive symptoms

Depressive symptoms were measured with the 11-item Centre for Epidemiologic Studies Depression Scale (CESD-11), a short version of the original 20-item CESD. The validity of the CESD-11 was confirmed in a previous Korean study.^28^ Each question was measured with a four-point Likert scale ranging from 0 to 3, so that the sum of the scores ranged from 0 to 33. The total score was multiplied by 20 and then divided by 11. We defined those with a score of ≥16 as having depressive symptoms, because the cut-off value of the 20-item CESD is 16.^29^

### Covariates

Gender, age, household income, education, marital status, occupation, weekly working hours, workplace size and survey year were considered as the confounders in our study. The age groups were classified as <30, 30–39, 40–49, 50–59 and ≥60 years. Household income was classified into quartiles (Q1–Q4). Education level was classified as having completed college, high school or below. Chronic disease status was classified as having at least one chronic disease being treated or taking medication or not. Smoking status was classified as current smokers or past/non-smokers. Marital status was classified as married, unmarried or other (divorced, widowed or separated). Occupation was classified as blue collar, service and sales worker, and white collar, according to the Korean Standard Classification of Occupations. The Korean Standard Classification of Occupations classifies managers, professionals and clerks as white-collar workers. Service workers and sales workers are classified as service and sales workers. Also, agricultural, forestry and fishery workers; craft and related trades workers; plant workers, machine operators and assemblers; and elementary workers are classified as blue-collar workers. Weekly working hours were classified as ≤40 or >40 h. According to the number of employees in the respondent's organisation, workplace size was classified as <30 workers, 30–99 workers or ≥100 workers. In addition, we also considered wave-specific effects by including binary dummy variables for each survey year in the fully adjusted models.

### Statistical analysis

For descriptive analysis, the *χ*^2^-test was used to compare the prevalence of alcohol use disorder and depressive symptoms according to the socioeconomic features of the study participants. For regression analysis, we initially performed pooled cross-sectional logistic regression and a generalised linear mixed model with a logit link in the next step to estimate the odds ratios and 95% confidence intervals. Models with random intercepts by individuals were used in the mixed-model analysis to analyse the repeatedly measured survey data. For model comparison, log likelihood and Akaike information criterion (AIC) values were calculated. For individual *i* and year *t*, the model specification is as follows:
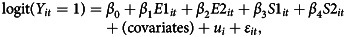


where *Y* denotes mental health outcomes (alcohol use disorder and depressive symptoms), *E*1 and *E*2 denote employment type (fixed-term workers and daily workers), *S*1 and *S*2 denote job satisfaction (neutral and dissatisfied), *u_i_* denotes participant-specific effect and ɛ*_it_* denotes the error. Also, to clarify the temporal association, we explored how the changes in employment status and job satisfaction affect mental health outcomes by applying a 1-year lag time to each independent variable.

For measuring the combined effect of temporary employment and job dissatisfaction, the two variables were grouped and newly classified into nine groups, ‘employment type (permanent worker) + job satisfaction (satisfied)’ to ‘employment type (daily labourer) + job satisfaction (dissatisfied)’. The relative excess risk due to interaction (RERI) was calculated to assess the supra-additive interaction between temporary employment and job dissatisfaction. We estimated the RERI by using the following equation:
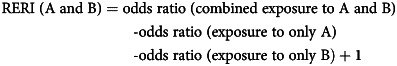


The 95% confidence interval of RERI was calculated by using the delta method.^30^ A RERI of >0 suggests supra-additive (synergistic) interaction between two variables. A *P*-value of <0.05 was considered statistically significant, and *P*-values were two-tailed. All statistical analyses were performed with R for Windows (version 4.2.1; R Foundation for Statistical Computing, Vienna, Austria; https://www.r-project.org). The R package glmmTMB was used to fit generalised linear mixed models.

## Results

Table 1 shows the socioeconomic features of the study participants based on pooled observations. Among the total observations, 29.7% and 13.5% were fixed-term workers and daily labourers, respectively. The highest prevalence of alcohol use disorder was observed among daily labourers (7.6%), followed by permanent workers (6.1%) and fixed-term workers (3.9%). The prevalence of depressive symptoms was 4.6% for permanent workers, 8.3% for fixed-term workers and 14.0% for daily labourers. Moreover, among those who were dissatisfied with their jobs, 8.7% and 18.2% of the workers had alcohol use disorder and depressive symptoms.

**Table 1:** Prevalence of alcohol use disorder and depressive symptoms by socioeconomic features among pooled observations

	Total	Alcohol use disorder			Depressive symptoms		
		Yes	No	*P*-value	Yes	No	*P-*value
		*N* = 2958 *n* (%)	*N* = 49 681 *n* (%)		*N* = 3673 *n* (%)	*N* = 48 966 *n* (%)	
Employment type							
Permanent worker	29 882 (56.8)	1815 (6.1)	28 067 (93.9)	<0.001	1374 (4.6)	28 508 (95.4)	<0.001
Fixed-term worker	15 632 (29.7)	602 (3.9)	15 030 (96.1)		1305 (8.3)	14 327 (91.7)	
Daily labourer	7125 (13.5)	541 (7.6)	6584 (92.4)		994 (14.0)	6131 (86.0)	
Job satisfaction							
Satisfied	32 038 (60.9)	1498 (4.7)	30 540 (95.3)	<0.001	1125 (3.5)	30 913 (96.5)	<0.001
Neutral	14 162 (26.9)	900 (6.4)	13 262 (93.6)		1373 (9.7)	12 789 (90.3)	
Dissatisfied	6439 (12.2)	560 (8.7)	5879 (91.3)		1175 (18.2)	5264 (81.8)	
Gender							
Male	28 270 (53.7)	2698 (9.5)	25 572 (90.5)	<0.001	1460 (5.2)	26 810 (94.8)	<0.001
Female	24 369 (46.3)	260 (1.1)	24 109 (98.9)		2213 (9.1)	22 156 (90.9)	
Age, years							
<30	8821 (16.8)	322 (3.7)	8499 (96.3)	<0.001	699 (7.9)	8122 (92.1)	<0.001
30–39	13 552 (25.7)	825 (6.1)	12 727 (93.9)		754 (5.6)	12 798 (94.4)	
40–49	15 849 (30.1)	1028 (6.5)	14 821 (93.5)		977 (6.2)	14 872 (93.8)	
50–59	10 970 (20.8)	654 (6.0)	10 316 (94.0)		901 (8.2)	10 069 (91.8)	
≥60	3447 (6.5)	129 (3.7)	3318 (96.3)		342 (9.9)	3105 (90.1)	
Education							
College	23 458 (44.6)	1142 (4.9)	22 316 (95.1)	<0.001	1197 (5.1)	22 261 (94.9)	<0.001
High school or lower	29 181 (55.4)	1816 (6.2)	27 365 (93.8)		2476 (8.5)	26 705 (91.5)	
Chronic disease							
No	30 099 (57.2)	2007 (5.5)	34 496 (94.5)	0.072	2162 (5.9)	34 341 (94.1)	<0.001
Yes	22 540 (42.8)	951 (5.9)	15 185 (94.1)		1511 (9.4)	14 625 (90.6)	
Smoking status							
Non/past smoker	39 155 (74.4)	1244 (3.2)	37 911 (96.8)	<0.001	2678 (6.8)	36 477 (93.2)	0.036
Current smoker	13 484 (25.6)	1714 (12.7)	11 770 (87.3)		995 (7.4)	12 489 (92.6)	
Household income (in quartile)							
1	13 165 (25.0)	400 (3.0)	12 765 (97.0)	<0.001	1578 (12.0)	11 587 (88.0)	<0.001
2	13 297 (25.3)	633 (4.8)	12 664 (95.2)		1036 (7.8)	12 261 (92.2)	
3	13 017 (24.7)	882 (6.8)	12 135 (93.2)		653 (5.0)	12 364 (95.0)	
4	13 160 (25.0)	1043 (7.9)	12 117 (92.1)		406 (3.1)	12 754 (96.9)	
Marital status							
Married	34 505 (65.6)	2103 (6.1)	32 402 (93.9)	<0.001	1837 (5.3)	32 668 (94.7)	<0.001
Unmarried	13 156 (25.0)	593 (4.5)	12 563 (95.5)		1070 (8.1)	12 086 (91.9)	
Other	4978 (9.5)	262 (5.3)	4716 (94.7)		766 (15.4)	4212 (84.6)	
Occupation							
Blue collar	21 089 (40.1)	1546 (7.3)	19 543 (92.7)	<0.001	1760 (8.3)	19 329 (91.7)	<0.001
Service and sales worker	9487 (18.0)	392 (4.1)	9095 (95.9)		772 (8.1)	8715 (91.9)	
White collar	22 063 (41.9)	1020 (4.6)	21 043 (95.4)		1141 (5.2)	20 922 (94.8)	
Weekly working hours							
≤40	30 099 (57.2)	1416 (4.7)	28 683 (95.3)	<0.001	2126 (7.1)	27 973 (92.9)	0.382
>40	22 540 (42.8)	1542 (6.8)	20 998 (93.2)		1547 (6.9)	20 993 (93.1)	
Workplace size							
<30 workers	27 507 (52.3)	1495 (5.4)	26 012 (94.6)	<0.001	2334 (8.5)	25 173 (91.5)	<0.001
30–99 workers	8479 (16.1)	422 (5.0)	8057 (95.0)		510 (6.0)	7969 (94.0)	
≥100 workers	16 653 (31.6)	1041 (6.3)	15 612 (93.7)		829 (5.0)	15 824 (95.0)	

Table 2 shows the association between temporary employment and job dissatisfaction, and alcohol use disorder. The fully adjusted generalised linear mixed model showed the best model fit (log likelihood and AIC). In the fully adjusted generalised linear mixed model, employment type and job satisfaction were associated with alcohol use disorder. Fixed-term workers did not show a significantly higher risk for alcohol use disorder (odds ratio 1.11, 95% CI 0.94–1.30) compared with permanent workers; however, being a daily labourer was associated with alcohol use disorder (odds ratio 1.54, 95% CI 1.22–1.95). Regarding job satisfaction, those who were neither satisfied nor dissatisfied (odds ratio 1.33, 95% CI 1.18–1.50) or were dissatisfied (odds ratio 1.78, 95% CI 1.52–2.08) with their jobs were associated with an increased risk for alcohol use disorder compared with those who were satisfied.

**Table 2:** Association of temporary employment and job dissatisfaction, and alcohol use disorder

	Pooled cross-sectional		Generalised linear mixed model	
	Crude model	Adjusted model	Crude model	Adjusted model
	Odds ratio [95% CI]	Odds ratio [95% CI]	Odds ratio [95% CI]	Odds ratio [95% CI]
Employment type				
Permanent worker	1.00 [Reference]	1.00 [Reference]	1.00 [Reference]	1.00 [Reference]
Fixed-term worker	0.57 [0.51–0.62]	1.07 [0.95–1.19]	0.84 [0.71–0.99]	1.11 [0.94–1.30]
Daily labourer	1.04 [0.94–1.15]	1.55 [1.35–1.77]	1.09 [0.86–1.37]	1.54 [1.22–1.95]
Job satisfaction				
Satisfied	1.00 [Reference]	1.00 [Reference]	1.00 [Reference]	1.00 [Reference]
Neutral	1.43 [1.31–1.56]	1.31 [1.19–1.43]	1.40 [1.23–1.59]	1.33 [1.18–1.50]
Dissatisfied	2.02 [1.81–2.24]	1.65 [1.47–1.85]	1.99 [1.69–2.35]	1.78 [1.52–2.08]
Gender				
Male		1.00 [Reference]		1.00 [Reference]
Female		0.17 [0.15–0.20]		0.13 [0.10–0.16]
Age, years				
<30		1.00 [Reference]		1.00 [Reference]
30–39		0.99 [0.85–1.15]		1.18 [0.94–1.49]
40–49		1.03 [0.88–1.21]		1.13 [0.87–1.48]
50–59		1.00 [0.84–1.19]		0.98 [0.72–1.32]
≥60		0.72 [0.57–0.92]		0.72 [0.48–1.06]
Household income (in quartile)				
1		1.00 [Reference]		1.00 [Reference]
2		1.34 [1.17–1.54]		1.32 [1.10–1.58]
3		1.51 [1.31–1.75]		1.50 [1.23–1.84]
4		1.78 [1.51–2.10]		1.81 [1.42–2.31]
Education				
College		1.00 [Reference]		1.00 [Reference]
High school or under		1.23 [1.11–1.35]		1.45 [1.18–1.79]
Chronic disease				
No		1.00 [Reference]		1.00 [Reference]
Yes		1.13 [1.04–1.24]		1.12 [0.99–1.27]
Smoking status				
Non/past smoker		1.00 [Reference]		1.00 [Reference]
Current smoker		2.00 [1.84–2.18]		2.34 [2.00–2.73]
Marital status				
Married		1.00 [Reference]		1.00 [Reference]
Unmarried		0.83 [0.74–0.94]		0.90 [0.71–1.13]
Other		1.09 [0.94–1.25]		1.05 [0.79–1.39]
Occupation				
Blue collar		1.00 [Reference]		1.00 [Reference]
Service and sales workers		1.06 [0.93–1.20]		1.07 [0.87–1.32]
White collar		1.12 [1.00–1.25]		1.10 [0.91–1.34]
Weekly working hours				
≤40		1.00 [Reference]		1.00 [Reference]
>40		1.11 [1.02–1.21]		1.16 [1.03–1.30]
Workplace size				
<30 workers		1.00 [Reference]		1.00 [Reference]
30–99 workers		0.92 [0.82–1.03]		0.95 [0.80–1.12]
≥100 workers		1.05 [0.95–1.17]		1.08 [0.92–1.28]
Log likelihood	−11220.26	−9933.17	−9055.685	−8642.778
Akaike information criterion	22450.53	19936.35	18123.37	17357.56

Table 3 shows the association between temporary employment and job dissatisfaction, and depressive symptoms. The fully adjusted generalised linear mixed model has the best model fit (log likelihood and AIC). In the generalised mixed model, employment type and job satisfaction were associated with depressive symptoms. Being a fixed-term worker (odds ratio 1.12, 95% CI 1.00–1.26) or daily labourer (odds ratio 1.68, 95% CI 1.44–1.95) was associated with depressive symptoms compared with permanent workers. Additionally, those who were neither satisfied nor dissatisfied (odds ratio 2.44, 95% CI 2.22–2.69) or were dissatisfied (odds ratio 4.88, 95% CI 4.36–5.46) with their jobs were associated with an increased risk for depressive symptoms.

**Table 3:** Association of temporary employment and job dissatisfaction, and depressive symptoms

	Pooled cross-sectional		Generalised linear mixed model	
	Crude model	Adjusted model	Crude model	Adjusted model
	Odds ratio [95% CI]	Odds ratio [95% CI]	Odds ratio [95% CI]	Odds ratio [95% CI]
Employment type				
Permanent worker	1.00 [Reference]	1.00 [Reference]	1.00 [Reference]	1.00 [Reference]
Fixed-term worker	1.53 [1.41–1.66]	1.11 [1.01–1.22]	1.48 [1.33–1.64]	1.12 [1.00–1.26]
Daily labourer	2.14 [1.96–2.34]	1.55 [1.38–1.75]	2.38 [2.10–2.70]	1.68 [1.44–1.95]
Job satisfaction				
Satisfied	1.00 [Reference]	1.00 [Reference]	1.00 [Reference]	1.00 [Reference]
Neutral	2.64 [2.43–2.87]	2.52 [2.31–2.74]	2.55 [2.32–2.81]	2.44 [2.22–2.69]
Dissatisfied	4.99 [4.56–5.46]	4.78 [4.35–5.26]	5.14 [4.60–5.74]	4.88 [4.36–5.46]
Gender				
Male		1.00 [Reference]		1.00 [Reference]
Female		2.01 [1.82–2.22]		2.17 [1.89–2.49]
Age				
<30		1.00 [Reference]		1.00 [Reference]
30–39		1.02 [0.90–1.16]		0.99 [0.84–1.16]
40–49		1.01 [0.88–1.17]		1.03 [0.86–1.24]
50–59		1.11 [0.95–1.29]		1.11 [0.90–1.36]
≥60		1.14 [0.94–1.37]		1.18 [0.93–1.51]
Household income (in quartile)				
1		1.00 [Reference]		1.00 [Reference]
2		0.78 [0.72–0.86]		0.76 [0.68–0.85]
3		0.68 [0.60–0.76]		0.70 [0.61–0.80]
4		0.63 [0.54–0.73]		0.62 [0.51–0.75]
Education				
College		1.00 [Reference]		1.00 [Reference]
High school or under		0.98 [0.90–1.08]		1.00 [0.88–1.14]
Chronic disease				
No		1.00 [Reference]		1.00 [Reference]
Yes		1.46 [1.35–1.58]		1.44 [1.31–1.58]
Smoking status				
Non/past smoker		1.00 [Reference]		1.00 [Reference]
Current smoker		1.56 [1.41–1.72]		1.60 [1.40–1.83]
Marital status				
Yes		1.00 [Reference]		1.00 [Reference]
No		1.49 [1.33–1.66]		1.47 [1.26–1.72]
Other		2.00 [1.81–2.21]		2.24 [1.92–2.61]
Occupation				
Blue collar		1.00 [Reference]		1.00 [Reference]
Service and sales workers		0.96 [0.87–1.06]		0.95 [0.84–1.09]
White collar		1.13 [1.01–1.26]		1.08 [0.94–1.24]
Weekly working hours				
≤40		1.00 [Reference]		1.00 [Reference]
>40		1.12 [1.03–1.21]		1.10 [1.00–1.20]
Workplace size				
<30 workers		1.00 [Reference]		1.00 [Reference]
30–99 workers		0.95 [0.85–1.05]		0.99 [0.87–1.12]
≥100 workers		1.13 [1.03–1.25]		1.11 [0.99–1.26]
Log likelihood	−12300.74	−11822.58	−11664.11	−11345.56
Akaike information criterion	24611.48	23715.16	23340.22	22763.13

**Fig. 1:**
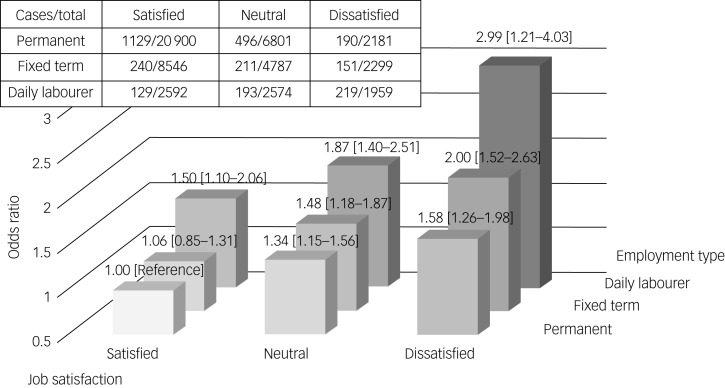
The combined effect of temporary employment and job dissatisfaction on alcohol use disorder, using a fully adjusted generalised linear mixed model.

**Fig. 2:**
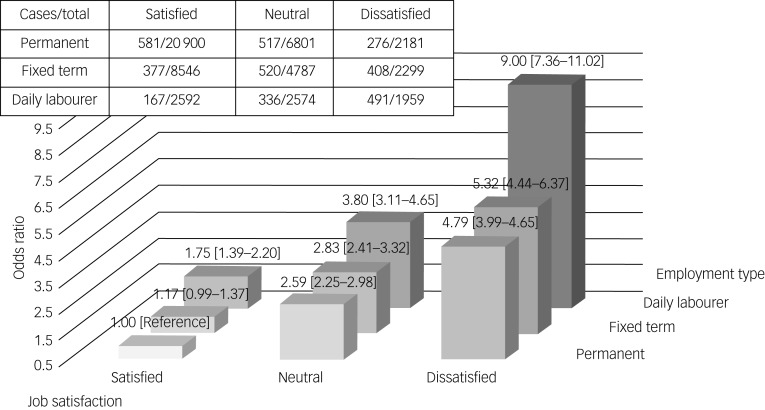
The combined effect of temporary employment and job dissatisfaction on depressive symptoms, using a fully adjusted generalised linear mixed model.

Figures 1 and 2 show the combined effect of temporary employment and job dissatisfaction on alcohol use disorder and depressive symptoms. This effect became stronger for those who were concurrently exposed to temporary employment and job dissatisfaction. For instance, daily labourers who were dissatisfied with their jobs showed the highest risk for alcohol use disorder (odds ratio 2.99, 95% CI 2.21–4.03) and depressive symptoms (odds ratio 9.00, 95% CI 7.36–11.02). The RERI was estimated to assess the additive interaction between temporary employment and job dissatisfaction. The RERI for fixed-term employment and job dissatisfaction was 0.36 (95% CI −0.26 to 0.99; *P* = 0.259) for alcohol use disorder and 0.37 (95% CI −0.69 to 1.42; *P* = 0.508) for depressive symptoms. The RERI for daily employment and job dissatisfaction was 0.91 (95% CI 0.06–1.76; *P* = 0.036) for alcohol use disorder and 3.47 (95% CI 1.80–5.14; *P* < 0.001) for depressive symptoms, suggesting a supra-additive interaction between the two variables.

Finally, Table 4 presents the effect of the sequential experience of employment status and job satisfaction (previous year → concerned year) on outcomes. The results show that the shift to daily employment (permanent/fixed-term → daily employment) and the retention of daily employment for two consecutive years (daily → daily employment) are significantly related to alcohol use disorders and depressive symptoms. In addition, deterioration of job satisfaction (satisfied/neutral → dissatisfied) and experience of job dissatisfaction for two consecutive years (dissatisfied → dissatisfied) are also related to alcohol use disorders and depressive symptoms.

**Table 4:** Association between changes in employment status and job satisfaction, and alcohol use disorder and depressive symptoms, using a fully adjusted generalised linear mixed model (*N* = 7481, observations: 39 107)

	Alcohol use disorder	Depressive symptoms
	Adjusted model	Adjusted model
	Odds ratio [95% CI]	Odds ratio [95% CI]
Change in employment type (year*_t–1_* → year*_t_*)		
Permanent/fixed term → permanent/fixed term	1.00 [Reference]	1.00 [Reference]
Daily → permanent/fixed term	1.17 [0.78–1.75]	1.18 [0.92–1.51]
Permanent/fixed term → daily	1.77 [1.15–2.71]	1.41 [1.06–1.88]
Daily → daily	1.94 [1.43–2.61]	1.92 [1.59–2.33]
Change in job satisfaction (year*_t–1_* → year*_t_*)		
Satisfied/neutral → satisfied/neutral	1.00 [Reference]	1.00 [Reference]
Dissatisfied → satisfied/neutral	1.05 [0.84–1.30]	1.68 [1.43–1.98]
Satisfied/neutral → dissatisfied	1.53 [1.24–1.88]	3.29 [2.83–3.82]
Dissatisfied → dissatisfied	1.71 [1.28–2.28]	3.85 [3.15–4.70]

## Discussion

Our study provides evidence of the detrimental health effects of temporary employment and job dissatisfaction. We investigated the effects of temporary employment and job dissatisfaction on alcohol use disorder and depressive symptoms. We confirmed that temporary employment and job dissatisfaction were associated with an increased risk of alcohol use disorder and depressive symptoms. Furthermore, our study suggests that the effects became stronger when temporary employment and job dissatisfaction were combined. In addition, the RERIs between daily employment and job dissatisfaction were >0 for alcohol use disorder and depressive symptoms, indicating a supra-additive (synergistic) interaction between the two variables.

Our findings are consistent with previous research showing that insecure employment is associated with alcohol use disorder^31,32^ and depressive symptoms,^9,33^ but contradict the finding that there is no significant association between temporary employment and alcohol use disorder.^13^ Although fixed-term employment was not associated with alcohol use disorder, daily labour was significantly associated with an increased risk of alcohol problems than permanent employment. In addition, daily labourers had higher odds ratios for alcohol use disorder and depressive symptoms compared with fixed-term workers, suggesting that a shorter duration of an employment contract is associated with an increased risk for mental health problems.

Along with temporary employment, our study also found that job dissatisfaction was significantly associated with alcohol use disorder and depressive symptoms. Our results support the findings from previous studies that alcohol use disorder and depressive symptoms are affected by workers’ job satisfaction.^18–20^ Additionally, the more that workers became dissatisfied with their job, the stronger the effect. For example, those who were dissatisfied with their job had higher odds ratios than those who were neither satisfied nor dissatisfied.

To the best of our knowledge, our study is the first to suggest that workers show greater risks for alcohol use disorder and depressive symptoms when they are simultaneously exposed to temporary employment and job dissatisfaction. According to previous studies, the job demand–control model^34^ and the effort–reward imbalance model^35^ explain workers’ stress. Job dissatisfaction reflects imbalances between job demand and control^36^ and job effort and reward.^37^ Additionally, insecure employment reduces workers’ perceived rewards, causing an imbalance between effort and reward.^38^ Thus, temporary employment and job dissatisfaction are more stressful when they are combined. Moreover, the combined effect of temporary employment and job dissatisfaction was greater than their sum.

Earlier studies have tried to identify the possible mechanisms by which temporary employment and job dissatisfaction affect workers’ mental health.^39,40^ The detrimental effect of temporary employment and job dissatisfaction on mental health problems could be explained by elevated work stress among employees. Employment insecurity and job dissatisfaction increase work-related stress^41^ and anxiety^19^ among workers, which in turn, increases the risk for mental health problems. One previous study revealed that stress-related mental health problems could be induced by job dissatisfaction.^39^ Similarly, Tatsuse et al found that lack of job satisfaction significantly predicted depression.^42^ Moreover, to cope with work stress triggered by job insecurity and dissatisfaction, employees may turn to risky health-related behaviours such as cigarette smoking, substance misuse and alcohol misuse.^40^ For example, stressful psychosocial work environment such as effort–reward imbalance can act as a trigger for heavy alcohol consumption.^43^

Physiological mechanisms also explain the relationship between temporary employment and job dissatisfaction, and mental health problems. According to previous studies, work stress and accumulated insecure employment induce dysregulation of the hypothalamic-pituitary-adrenal (HPA) axis.^44,45^ Overactivation of the HPA axis is closely related to heavy alcohol consumption^46^ and major depression.^47^ Thus, dysregulation of the HPA axis may mediate the relationship between temporary employment and job dissatisfaction, and alcohol use disorder and depressive symptoms. Further in-depth studies are needed to elucidate the exact mechanism of the effects of temporary employment and job dissatisfaction on workers’ mental health.

Our study has some limitations. First, the KoWePS questionnaire regarding job dissatisfaction measured workers’ overall job satisfaction. Thus, detailed aspects of the work environment, such as job demand and control, hazardous physical and psychological threats, and perceived insecurities, could not be identified in this study because of a lack of information. Second, as health outcomes in our study were self-reported, social desirability bias and recall bias should be considered. One previous study reported that survey respondents tended to underreport their alcohol consumption.^48^ Thus, there is a possibility that the overall prevalence of alcohol use disorder might be underestimated in this study. Third, although we employed a generalised linear mixed model to investigate the association between job characteristics and mental health problems, the possibility of reverse causality should be explored in future studies. For example, the mental health problems of workers could negatively affect their employment status^49^ and job satisfaction.^18^

Despite these limitations, our study has several strengths. First, we used mixed models adjusted for the personal traits of the survey participants. Personal traits can confound the relationship between job satisfaction and mental health problems. For example, workers with high levels of neuroticism have been known to report more job dissatisfaction and mental health problems.^50^ From this perspective, our study overcame the limitations of previous cross-sectional studies. Next, the KoWePS study participants were selected through a systemic sampling method; therefore, the participants represented households in Korea. Thus, our study has strength in terms of its generalisability.

In summary, our current study found that temporary employment and job dissatisfaction have detrimental effects on alcohol use disorder and depressive symptoms. These effects became stronger when temporary employment and job dissatisfaction were combined. Policy makers should implement appropriate actions to ease job insecurity among temporarily employed workers, and to improve job satisfaction.

## Data Availability

The raw Korea Welfare Panel Study (KoWePS) data are available online (https://www.KoWePS.re.kr:442/main.do). The raw KoWePS data do not comprise personal information.
